# Circadian ATP Release in Organotypic Cultures of the Rat Suprachiasmatic Nucleus Is Dependent on P2X7 and P2Y Receptors

**DOI:** 10.3389/fphar.2018.00192

**Published:** 2018-03-06

**Authors:** Irena Svobodova, Anirban Bhattaracharya, Milorad Ivetic, Zdenka Bendova, Hana Zemkova

**Affiliations:** ^1^Department of Cellular and Molecular Neuroendocrinology, Institute of Physiology of the Czech Academy of Sciences, Prague, Czechia; ^2^Department of Physiology, Faculty of Science, Charles University, Prague, Czechia

**Keywords:** suprachiasmatic nucleus, organotypic cultures, astrocytes, P2X7 receptor, P2Y1 receptor, P2Y2 receptor, pannexin-1 hemichannel, ATP release

## Abstract

The circadian rhythms in physiological and behavioral functions are driven by a pacemaker located in the suprachiasmatic nucleus (SCN). The rhythms continue in constant darkness and depend on cell-cell communication between neurons and glia. The SCN astrocytes generate also a circadian rhythm in extracellular adenosine 5′-triphosphate (ATP) accumulation, but molecular mechanisms that regulate ATP release are poorly understood. Here, we tested the hypothesis that ATP is released via the plasma membrane purinergic P2X7 receptors (P2X7Rs) and P2Y receptors (P2YRs) which have been previously shown to be expressed in the SCN tissue at transcriptional level. We have investigated this hypothesis using SCN organotypic cultures, primary cultures of SCN astrocytes, ATP bioluminescent assays, immunohistochemistry, patch-clamping, and calcium imaging. We found that extracellular ATP accumulation in organotypic cultures followed a circadian rhythm, with a peak between 24:00 and 04:00 h, and the trough at ~12:00 h. ATP rhythm was inhibited by application of AZ10606120, A438079, and BBG, specific blockers of P2X7R, and potentiated by GW791343, a positive allosteric modulator of this receptor. Double-immunohistochemical staining revealed high expression of the P2X7R protein in astrocytes of SCN slices. PPADS, a non-specific P2 antagonist, and MRS2179, specific P2Y1R antagonist, also abolished ATP rhythm, whereas the specific P2X4R blocker 5-BDBD was not effective. The pannexin-1 hemichannel blocker carbenoxolone displayed a partial inhibitory effect. The P2Y1R agonist MRS2365, and the P2Y2R agonist MRS2768 potentiated ATP release in organotypic cultures and increase intracellular Ca^2+^ level in cultured astrocytes. Thus, SCN utilizes multiple purinergic receptor systems and pannexin-1 hemichannels to release ATP.

## Introduction

The suprachiasmatic nucleus (SCN) of the anterior hypothalamus is the principal circadian pacemaker in mammals (Moore and Eichler, 1972; Stephan and Zucker, 1972). In most species, including rat, the SCN has two subdivisions that differ in neuronal input and neuropeptide contents. The ventrolateral part of the SCN receives glutamatergic inputs from retinal ganglion cells and produces vasoactive intestinal polypeptide, whereas the dorsomedial part does not receive a direct visual input and produces arginine vasopressin (AVP) (Moore and Card, 1985; Jacomy et al., 1999). Circadian rhythm in AVP secretion parallels rhythm of electrical activity in SCN neurons (Inouye and Kawamura, 1979; Groos and Hendriks, 1982; Pennartz et al., 2002) and both these rhythms are mediated by the expression of clock genes (Reppert, 1998). The SCN generates also a circadian rhythm in extracellular adenosine 5′-triphosphate (ATP) accumulation, which negatively correlates with the electrical activity and AVP secretion rhythms (Yamazaki et al., 1994; Womac et al., 2009). Extracellular ATP levels fluctuate rhythmically within the rat SCN *in vivo* and SCN-derived SCN2.2 cell cultures containing 80% astrocytes (Womac et al., 2009; Burkeen et al., 2011), indicating that oscillations in ATP release are intrinsic properties of SCN astrocytes. However, the pathway by which ATP travels from the cytosol of SCN cells to the extracellular space is still unknown.

ATP release from astrocytes was originally thought to be vesicular and dependent on Ca^2+^ (Fumagalli et al., 2003; Pascual et al., 2005; Pangrsic et al., 2007). Consistent with this hypothesis, large amounts of ATP have been detected in the dense-core vesicles and lysosomes of astrocytes (Hong et al., 2016). Changes in intracellular Ca^2+^ levels in rat SCN2.2 cells, however, are inversely related to the circadian variations in extracellular ATP accumulation (Burkeen et al., 2011). Moreover, genetic disruption of the vesicular release mechanism had no effect on circadian ATP release in cultured mouse cortical astrocytes (Marpegan et al., 2011), indicating that circadian ATP release might occur via a non-vesicular pathway, possibly through conductive mechanisms involving pore-forming molecules such as pannexin-1 hemichannels (Stout et al., 2002; Schenk et al., 2008; Iglesias et al., 2009; Li S. et al., 2011) or purinergic P2X7 receptor (P2X7R) channel (Khakh and Sofroniew, 2015). P2X7R is ATP-gated ion channel (Surprenant et al., 1996) that can form a large pore itself (Khakh and Lester, 1999) or after interaction with another transmembrane molecule (Pelegrin and Surprenant, 2006; Locovei et al., 2007). The P2X7R has been detected in astrocytes (Narcisse et al., 2005; Sperlágh et al., 2006; Hamilton et al., 2008) and complexes with pannexin to promote Ca^2+^-independent gliotransmitter release (Ballerini et al., 1996; Wang et al., 2002; Duan et al., 2003; Hamilton et al., 2008; Carrasquero et al., 2009; Nörenberg et al., 2011b). As shown in our previous RT-PCR analysis, P2X7R is the second most highly expressed P2X subunit in the SCN after the P2X2R which are localized on presynaptic nerve terminals in the SCN (Bhattacharya et al., 2013). In addition to transcripts for P2X receptors, several P2Y receptors (P2Y1 and P2Y2) have also been identified in the SCN (Bhattacharya et al., 2013). Thus, in this work, we tested the hypothesis that P2X7Rs and P2YRs are associated with circadian ATP release from SCN astrocytes. We have investigated this hypothesis using organotypic cultures of rat brain slices containing the SCN, primary cultures of SCN astrocytes, ATP bioluminescent assays, immunohistochemistry, patch-clamping, and calcium imaging.

## Materials and methods

### Animals and brain slices

The Animal Care and Use Committee of the Czech Academy of Sciences approved the experiments of the present study. Experiments were performed in Wistar rats of both genders, 16- to 21-days-old, which were kept under a controlled 12–12-h light-dark cycle from birth with food and water available *ad libitum*. Animals had lights on from 6 a.m. to 6 p.m. Brains were removed after decapitation and placed into ice-cold (4°C) oxygenated (95% O_2_ + 5% CO_2_) artificial cerebrospinal fluid (ACSF) that contained: 130 mM NaCl, 3 mM KCl, 1 mM MgCl_2_, 2 mM CaCl_2_, 19 mM NaHCO_3_, 1.25 mM NaH_2_PO_4_ and 10 mM glucose (pH 7.3–7.4; osmolality 300–315 mOsm).

### Organotypic culture preparation

Coronal sections of the hypothalamus (~350 μm thick) were cut from ~1 × 1 mm tissue blocks containing the SCN using a vibratome (DTK-1000, D.S.K. Dosaka, Japan). In some experiments, the SCN was punctured out from slices. Three slices were cut from one animal and then transferred onto one cell culture insert with a pore size of 0.001 mm (BD Falcon, Tewksbury, MA, USA). Culture inserts with 3 slices, further referred to as the organotypic cultures, were placed in 6-well plates (BD Falcon) and submerged in 1 ml of Neurobasal A medium supplemented with 2% serum-free B-27, 50 U/ml penicillin, 50 μg/ml streptomycin and 0.5 mM L-glutamine (all from Thermo Fisher Scientific, Waltham, MA) saturated with a 95% air and 5% CO_2_ mixture. Plates containing inserts with slices/cultures were incubated in a humidified 5% CO_2_ atmosphere at 37°C. Slices were cultured in Neurobasal medium for 7 days before starting ATP accumulation assays to allow slices to stabilize.

### Acute slices

Hypothalamic slices (200–300 μm thick) containing SCN were incubated as described previously (Kretschmannova et al., 2003; Vavra et al., 2011; Bhattacharya et al., 2013). Briefly, the slices were allowed to recover for at least 1 h in oxygenated ACSF at 32–33°C before being transferred into a recording chamber. During the experiments, slices were fixed with a platinum U-shaped wire to the bottom of the chamber and submerged in continuously flowing oxygenated ACSF at 1–2 ml min^−1^ at room temperature. Slices were viewed with an upright microscope (Olympus BX50WI, Melville, NY, USA) mounted on a Gibraltar movable X-Y platform (Burleigh) using water immersion lenses (60x and 10x) and Dodt infrared gradient contrast (Luigs & Neumann, GmbH, Germany). SCN regions were identified by their position relative to the third ventricle and the optic chiasm in a coronal hypothalamic section as described previously (Kretschmannova et al., 2003; Bhattacharya et al., 2013).

### Primary cultures of SCN astrocytes

Postnatal day 2–5 newborn rats were euthanized by decapitation. SCN regions were dissected from ~600 μm thick hypothalamic slices and cells were dissociated after treatment with trypsin, according to published methods (Watanabe et al., 1993; Bhattacharya et al., 2013). Next, cells were purified on a discontinuous protein gradient, and ~100,000 cells were placed on coverslips coated with a 1% poly-L-lysine solution (Sigma) in 35 mm culture dishes (BD Falcon) and cultured in Neurobasal A medium with 2% B27 supplement and 0.5 mM L-Glutamine in a humidified CO_2_-containing atmosphere at 37°C until use (14–21 days).

### Measurement of ATP production

ATP secretion by SCN cells into the medium from the above organotypic cultures was measured every 4 h over a 24–48 h incubation period. Extracellular ATP concentrations in the medium were determined using an ATP bioluminescent assay. Before assay, at 8:00 h, cultures were washed with ATP-free Dulbecco's Modified Eagle's Medium (DMEM; Thermo Fisher Scientific, Waltham, MA) supplemented with 50 U/ml penicillin and 50 μg/ml streptomycin, and then incubated with fresh ATP-free DMEM-based medium in a humidified 5% CO_2_ atmosphere at 37°C for 4 h. In our experimental protocol spanning 24–48 h, media (1 ml) above the slices were collected every 4 h starting at 12:00 h. Samples were collected as a full volume of medium (1 ml) and were replaced with fresh ATP-free DMEM. Media samples were then stored at −20°C for 3 days, and the ATP concentration was then measured using an ATP Bioluminescence Assay Kit CLS II (Hoffmann-La Roche AG, Basel, Switzerland).

Treatments were performed by completely replacing the medium with fresh drug-containing culture medium every 4 h. Each tested drug was added at 8:00 h, and the cultures were exposed to these drugs over a 24–48 h incubation period. In control experiments, the protocol was identical and the medium was replaced with fresh drug-free culture medium every 4 h.

### Immunohistochemistry

Adult male rats were deeply anesthetized with thiopental (50 mg/kg) and perfused through the aorta with heparinized saline followed by phosphate-buffered saline (PBS; 0.01 M sodium phosphate/0.15 M NaCl, pH 7.2) and 4% paraformaldehyde in PBS. Brains were removed, postfixed for 12 hrs at 4°C, cryoprotected in 20% sucrose in PBS overnight at 4°C, and stored at −80°C. Brains were sectioned into series of 30-μm-thick free-floating coronal slices throughout the rostral-caudal extent of the SCN. Levels of P2X7 receptor protein in astrocytes were assessed using anti-GFAP conjugated with Cy3 (ab49874, Abcam, Cambridge, United Kingdom) mouse monoclonal, 1:1,000, anti-P2X7 (APR-004, Alomone Labs, Israel) rabbit, 1:1,000. The P2X7 labeling was visualized using Alexa Fluor® 488-conjugated secondary anti-rabbit antibody (Invitrogen, Carlsbad, CA; dilution 1:500). The images were acquired with confocal microscope Leica TCS SP2.

### Calcium imaging

For intracellular Ca^2+^ fluorescence imaging, primary 14-21 days-old SCN cultures, that were enriched in astrocytes, were incubated in 2 ml ACSF containing 1 μM of membrane-permeant ester form of Fura-2 (Fura-2AM, Invitrogen, Molecular Probes) and 0.15% dispersing agent Pluronic F-127 for 30–45 min in carbogen atmosphere (95% O2 and 5% CO2). After 15 min of washing in fresh ASCF, Fura-2 fluorescence from single cells was measured using a MicroMAX CCD camera (Princenton Instruments, USA) and an Olympus BX50WI epifluorescent microscope coupled to a monochromatic illumination system (T.I.L.L., Photonics). Hardware control and image analysis were performed using MetaFluor software (Molecular Devices). Cells were examined under a water immersion objective during exposure to alternating 340- and 380-nm light beams. The emitted light images at 515 nm were acquired through a d 40 × 0.9 NA objective, and the intensity of light emission was measured. The ratio of light intensity (F_340_/F_380_) reflects changes in intracellular free Ca^2+^ concentration ([Ca^2+^]_i_) and was followed in ~20 single cells simultaneously at the rate of one point per second.

### Patch clamp recordings

Action potentials were recorded from SCN neurons in acutely isolated slices using standard whole-cell patch clamp techniques with an Axopatch-200B amplifier (Molecular Devices, Sunnyvale, CA, USA). Patch pipettes were pulled on the horizontal Flaming Brown P-97 model puller (Sutter Instruments, Novato, CA, USA) from borosilicate glass (World Precision Instruments, Sarasota, FL) and polished by heat to a tip resistance of 4–6 MΩ. Data were captured and stored using the pClamp 10 software package in conjunction with the Axon^TM^ Digidata® 1550A A/D converter (Molecular Devices). Signals were filtered at 1 kHz and sampled at 2 kHz. Drugs were diluted and applied in a N-2-hydroxyethylpiperazine-N′-2-ethanesulfonic acid (HEPES)-buffered extracellular solution containing: 142 mM NaCl, 3 mM KCl, 1 mM MgCl_2_, 2 mMCaCl_2_, 10 mM glucose and 10 mM HEPES, pH was adjusted to 7.3 with 1 M NaOH; osmolality was 300–315 mOsm. Patch electrodes used for whole-cell recording were filled with an intracellular solution containing: 140 mM KCl, 3 mM MgCl_2_, 0.5 mM CaCl_2_, 10 mM HEPES, and 5 mM EGTA; pH was adjusted to 7.2 with KOH. The osmolality of the intracellular solutions was 285–295 mOsm. ATP and drugs were applied in HEPES-buffered extracellular solution delivered to the recorded cells by a gravity-driven microperfusion system containing nine glass tubes with a common outlet of ~300 μM in diameter. The application tip was routinely positioned ~500 μm away from the recorded cell and ~50 μm above the surface of the slice.

### Experimental design and statistical analysis

Each experiment was performed on 12 independent organotypic cultures prepared from 12 animals (3 control and 3 × 3 experimental, drug-treated, cultures). Each study was repeated on cultures derived from 3 to 7 different dissections. ATP release in each experiment represents a mean of ATP release from three cultures. Throughout the manuscript, “*n*” is defined as the numbers of independent experiments. Cumulated ATP secretion was calculated as a sum of all 4 h data points obtained from each of the incubated cultures at the end of incubation period. Statistical comparison of multiple groups was made by using one-way analysis of variance in SigmaStat 2000 v9.0, followed by Tukey's *post-hoc* test for comparison to a single control, or the Student's *t*-test for comparison between two groups (^**^p < 0.01 and ^*^p < 0.05). Graphing was performed using SigmaPlot (Systat Software) and CorelDraw (Corel Corporation) software. All values are reported as the means ± SEM and sample sizes are *n* = 3–7.

### Chemicals

3-[[5-(2,3-dichlorophenyl)-1*H*-tetrazol-1-yl]methyl] pyridine hydrochloride (A438079); Apyrase; D-2-Amino-5-phosphonopentanoic acid (AP5); *N*-Cyano-*N*”-[(1*S*)-1-phenylethyl]-*N*'-5-quinolinyl-guanidine (A804598);*N*-[2-[[2- [(2–Hydroxyethyl)amino]ethyl]amino]-5-quinolinyl]-2-tricyclo[3.3.1.13,7]dec-1-ylacetamide dihydrochloride (AZ10606120); 5-(3-Bromophenyl)-1,3-dihydro-2*H*-benzofuro[3,2-*e*]-1,4-diazepin-2-one (5-BDBD); 7-Chloro-5-(2-chlorophenyl)-1,5-dihydro-4,1-benzothiazepin-2(3*H*)-one (CGP37157); 2-[(3,4-Difluorophenyl)amino]-*N*-[2-methyl-5-(1-piperazinylmethyl)phenyl]-acetamide trihydrochloride (GW791343); pyridoxalphosphate-6-azophenyl-2′, 4′-disulfonic acid (PPADS); 2-deoxy-*N*6-methyladenosine 3,5-bisphosphate tetrasodium salt (MRS2179); [[(1*R*,2*R*,3*S*,4*R*,5*S*)-4-[6-Amino-2-(methylthio)-9*H*-purin-9-yl]-2,3-dihydroxybicyclo[3.1.0]hex-1-yl]methyl] diphosphoric acid mono ester trisodium salt (MRS2365) and Uridine-5′-tetraphosphate δ-phenyl ester tetrasodium salt (MRS2768) were purchased from Tocris-Cookson (Bristol, UK). Brilliant Blue G (BBG); 2′-3′-O-(4-benzoylbenzoyl)-ATP (BzATP); carbenoxolone (CBX); nifedipine and all other drugs and chemicals were from Sigma (St. Louis, MO). The A438079, AZ10606120, 5-BDBD, CGP37157, nifedipine and PPADS were used at 1 nM−10 μM from a 10 mM working stocks in DMSO; hence, 0.00001–0.1% (v/v) DMSO vehicle controls were applied in parallel to these incubations.

## Results

### Characterization of the circadian ATP release in SCN organotypic cultures

**Figure 1:**
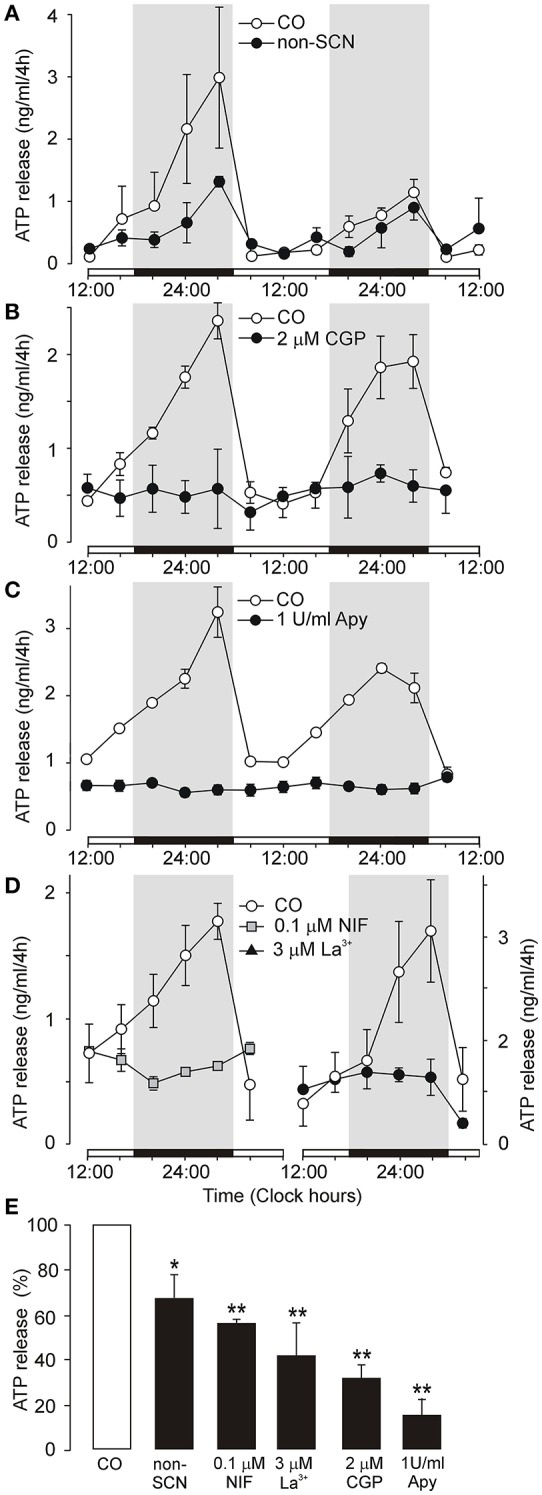
Endogenous circadian oscillations of ATP release in organotypic cultures of the rat SCN. **(A–C)** Examples of an ATP rhythm measured during 2 days in control cultures (open symbols) and experimental cultures (closed symbols). Comparison of ATP rhythm in control organotypic cultures containing the SCN (CO) and organotypic cultures lacking the SCN (non-SCN) **(A)**. Inhibition of ATP release by a selective inhibitor of Na^+^-Ca^2+^ exchange in mitochondria, the benzothiazepine CGP37157 (CGP) **(B)**, basal ATP release in the presence of ecto-ATPase, apyrase (Apy) **(C)** and inhibition of ATP release by the L-type Ca^2+^ channel inhibitor nifedipine (**D**, left panel) and a non-selective voltage-gated Ca^2+^ channel blocker La^3+^ (**D**, right panel). Summary histogram showing cumulated ATP release at the end of the incubation period in control cultures (open column) and experimental cultures (black columns) **(E)**. Medium was sampled every 4 h and the ATP content was quantified (in ng/ml). ATP release was spontaneous, with a circadian rhythm that is a continuation of the endogenous rhythm in the SCN *in vivo*. Open horizontal bars on X axes thus indicate the light periods experienced by donor animals (from 12:00 to 24:00 h and from 24:00 to 12:00 h) and solid bars indicate the dark periods (12 h). Treatments were performed by completely replacing the medium with fresh drug-containing culture medium every 4 h throughout the whole incubation period. Tested drugs were applied at 08:00 h and measurements started at 12:00 h. Parallel cultures from the same experiments are shown. Data are presented as the means ± SEM, each study was repeated on cultures derived from 3 to 6 different dissections (experiments). Statistical significance of differences between control and experimental groups: ^*^P < 0.05 and ^**^P < 0.01.

Organotypic SCN cultures maintain the clear organization of SCN cells along the dorsoventral axis (Figure S1) and exhibit circadian rhythm in secretion of AVP for up to 2 weeks *in vitro*, with a phase that is consistent with the light regime experienced by the donor animal (Svobodova et al., 2003). Our present study shows that ATP accumulation in the medium also followed a circadian rhythm: the peak occurred between 24:00 – 04:00 h, and the trough occurred at ~12:00 h (Figures 1A–D, open circles). The time points of the peak and trough were stable in 62 of 66 independent control cultures (94%). In the remaining 6% of cultures (*n* = 4), either no rhythm was observed or the peak did not occur between 24:00 and 04:00 h; these cultures were discarded. These data show that circadian rhythm of ATP release peaks at an opposite phase to the previously described AVP secretory rhythm that peaked at ~12:00 h in similar organotypic SCN cultures (Svobodova et al., 2003).

We monitored extracellular ATP accumulation in organotypic cultures from which the SCN was removed to determine the role of the SCN. In non-SCN cultures, the circadian rhythm of extracellular ATP accumulation was still present (Figure 1A), but exhibited lower robustness, and cumulated ATP release was significantly reduced compared to control SCN-containing cultures (66 ± 11%, *n* = 3, *P* < 0.05; Figure 1E). Thus, in addition to the SCN, other hypothalamic nuclei located near the SCN that also express components of the molecular clock, such as the lateral hypothalamus and supraoptic nucleus (Abe et al., 2002; Guilding et al., 2009), contribute to circadian ATP release rhythms but with a lower efficacy as compared to the SCN.

Next, we performed a bath application of selective inhibitor of the mitochondrial Na^+^-Ca^2+^ exchange transporter CGP37157 (White and Reynolds, 1997) to examine a possible link between extracellular ATP accumulation and mitochondrial function (Burkeen et al., 2011). The effective threshold concentration of CGP37157 that inhibited ATP release was 200 nM (43 ± 8% of control, *n* = 3, *P* < 0.01; not shown), and a 2 μM treatment inhibited ATP release to 31 ± 6% of control (*n* = 4; Figure 1B). Incomplete inhibition of extracellular ATP accumulation after treatment with CGP37157 indicates that ATP is released as a transmitter, not as a metabolite.

We also wanted to determine basal ATP release that persists after ATP hydrolysis. *In vivo*, the ecto-nucleotide triphosphate diphosphohydrolase family of enzymes (eNTPDases) hydrolyze extracellular ATP and/or ADP to AMP and adenosine (Zimmermann, 2000). The addition of apyrase (1 U/ml), a soluble ecto-nucleotidase, completely abolished the ATP secretory rhythm (Figure 1C). However, apyrase did not remove all ATP, as basal ATP concentrations were observed in the bath (14 ± 8% of control, *n* = 3; Figure 1E), most probably reflecting ATP contained in a compartment not accessible to apyrase such as exosomes. Adenosine (100 nM) was not effective inducer of ATP release (124 ± 15% of control, *n* = 3; Figure S2), suggesting that endogenous ATP does not act at adenosine P1 receptors following extracellular metabolism.

To examine the dependence on extracellular Ca^2+^, we tested the effect of voltage-gated Ca^2+^ channel blockers. Consistent with a hypothesis that astrocytes release ATP by Ca^2+^-dependent mechanism (Fumagalli et al., 2003) and express voltage-gated calcium channels (Yan et al., 2013), nifedipine (0.1 μM), the L-type Ca^2+^ channel inhibitor, and La^3+^ (3 μM), a non-selective voltage-gated Ca^2+^ channel blocker, abolished ATP rhythm and significantly inhibited cumulated ATP release (nifedipine: 56 ± 3% of the control, *n* = 3, *P* < 0.05; La^3+^: 41 ± 15% of the control, *n* = 3, *P* < 0.01, Figures 1D,E). Since Ca^2+^ channel blockers significantly, but not completely inhibited ATP release, these results indicate that Ca^2+^ -independent mechanism(s) might also play a role in evoking ATP release.

### Dependence of extracellular ATP accumulation on P2X7R activity

**Figure 2:**
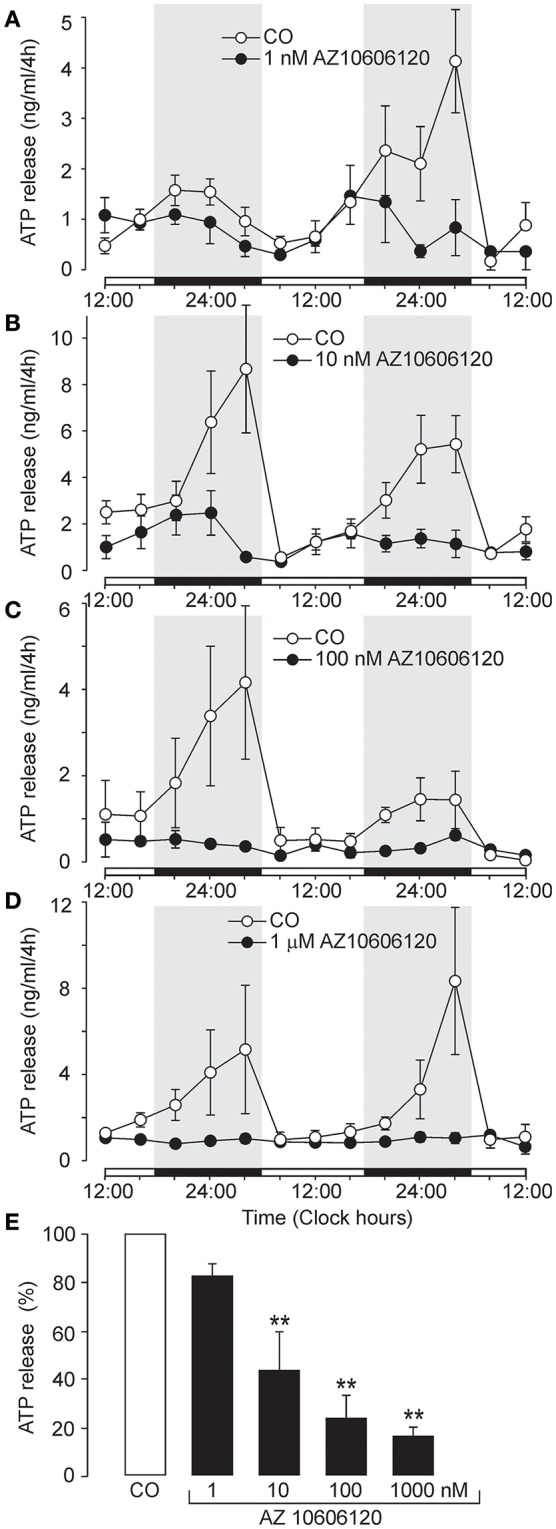
Inhibition of circadian ATP release by the selective P2X7R blocker AZ10606120. **(A–D)** Examples of the ATP release rhythm in control cultures (open symbols) and cultures incubated with the P2X7R negative allosteric modulator AZ10606120 (closed symbols): 1 nM **(A)**, 10 nM **(B)**, 100 nM **(C)**, and 1 μM **(D)**. Summary histogram comparing the effects of various concentrations of AZ10606120 on cumulated ATP release **(E)**. Data are presented as the means ± SEM of three to six experiments. ^**^P < 0.01 compared to the control.

**Figure 3:**
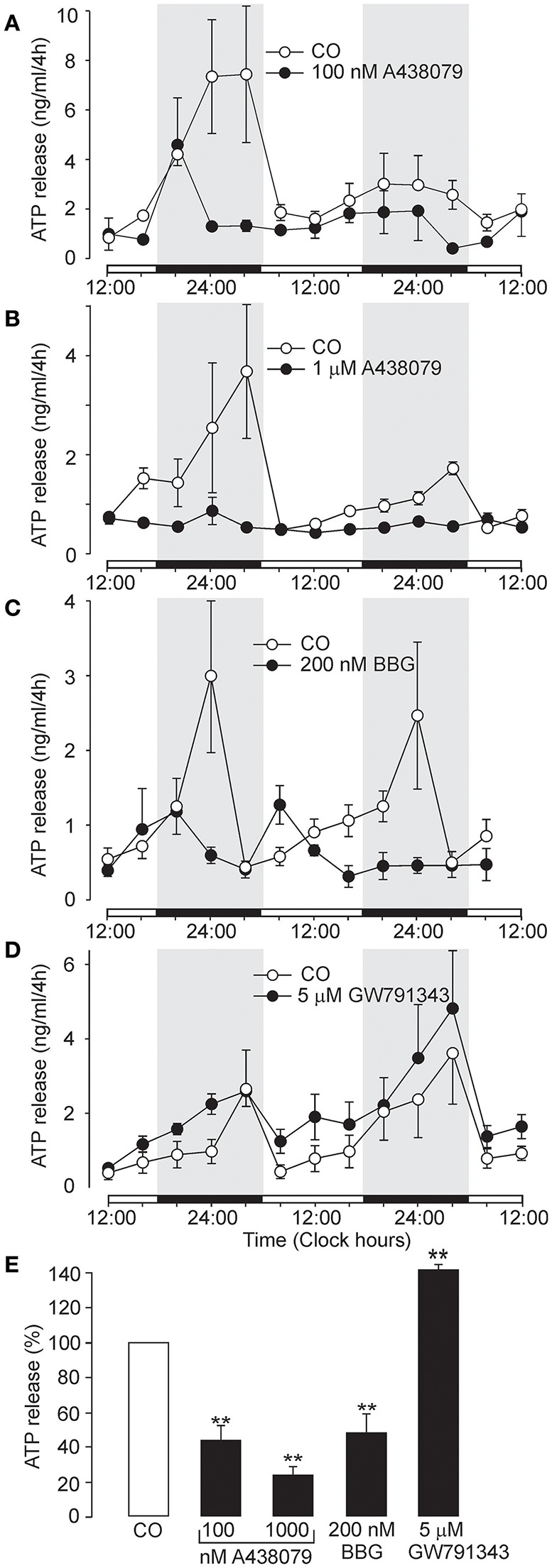
Inhibition of circadian ATP release by the selective P2X7R inhibitors A438079 and BBG, and potentiation by positive allosteric modulator GW791343. **(A–D)** Examples of the ATP release rhythm in control cultures (open symbols) and cultures treated with selective P2X7R modulators (closed symbols). Inhibitory effects of 100 nM A438079 **(A)**, 1 μM A438079 **(B)**, and 200 nM BBG **(C)**, and potentiating effect of the positive allosteric modulator GW791343 at 5 μM concentration **(D)**. Summary graph comparing the effects of positive and negative modulators on cumulated ATP **(E)**. Data are presented as the means ± SEM of three to seven experiments. ^**^P < 0.01 as compared to the control.

We examined the effects of several selective P2X7R antagonists to investigate the role of P2X7R in ATP release from SCN organotypic cultures (Figures 2, 3). At low concentrations (1–10 nM), negative allosteric modulator AZ10606120 (Michel et al., 2007) reduced the amplitude of the circadian ATP rhythm (Figures 2A,B) and partially inhibited cumulated ATP release (10 nM: 44 ± 16% of the control, *n* = 3, *P* < 0.01; Figure 2E). At higher concentrations (100 nM–1 μM), AZ10606120 completely inhibited extracellular ATP rhythm (Figures 2C,D) to approximately basal levels (100 nM: 24 ± 7% of the control, *n* = 4, *P* < 0.01; 1 μM: 16 ± 3% of the control, *n* = 3, *P* < 0.01; Figure 2E), similar to levels observed in the presence of apyrase (Figure 1C).

Another selective P2X7R antagonist, the structurally unrelated negative allosteric modulator A438079 (Donnelly-Roberts et al., 2009), also inhibited ATP release in a concentration-dependent manner (Figures 3A,B). The effective threshold concentration was 10 nM (data not shown), and at concentrations of 100 nM and 1 μM, ATP release was inhibited to 44 ± 7% (*n* = 4, *P* < 0.01) and 30 ± 5% (*n* = 3, *P* < 0.01) of the control level, respectively (Figure 3E). Treatment with the classical P2X7R antagonist Brilliant Blue G (BBG; 200 nM) (Jiang et al., 2000) abolished the ATP rhythm (Figure 3C) and inhibited ATP release at the end of the incubation period to 48 ± 11% of control levels (*n* = 3, *P* < 0.01; Figure 3E). In contrast GW791343 (100 nM), a positive allosteric modulator of rat P2X7R (Michel et al., 2008), enhanced the amplitude of ATP release rhythm (Figure 3D) and extracellular ATP accumulation to 144 ± 6% of control levels (*n* = 3, *P* < 0.01; Figure 3E).

**Figure 4:**
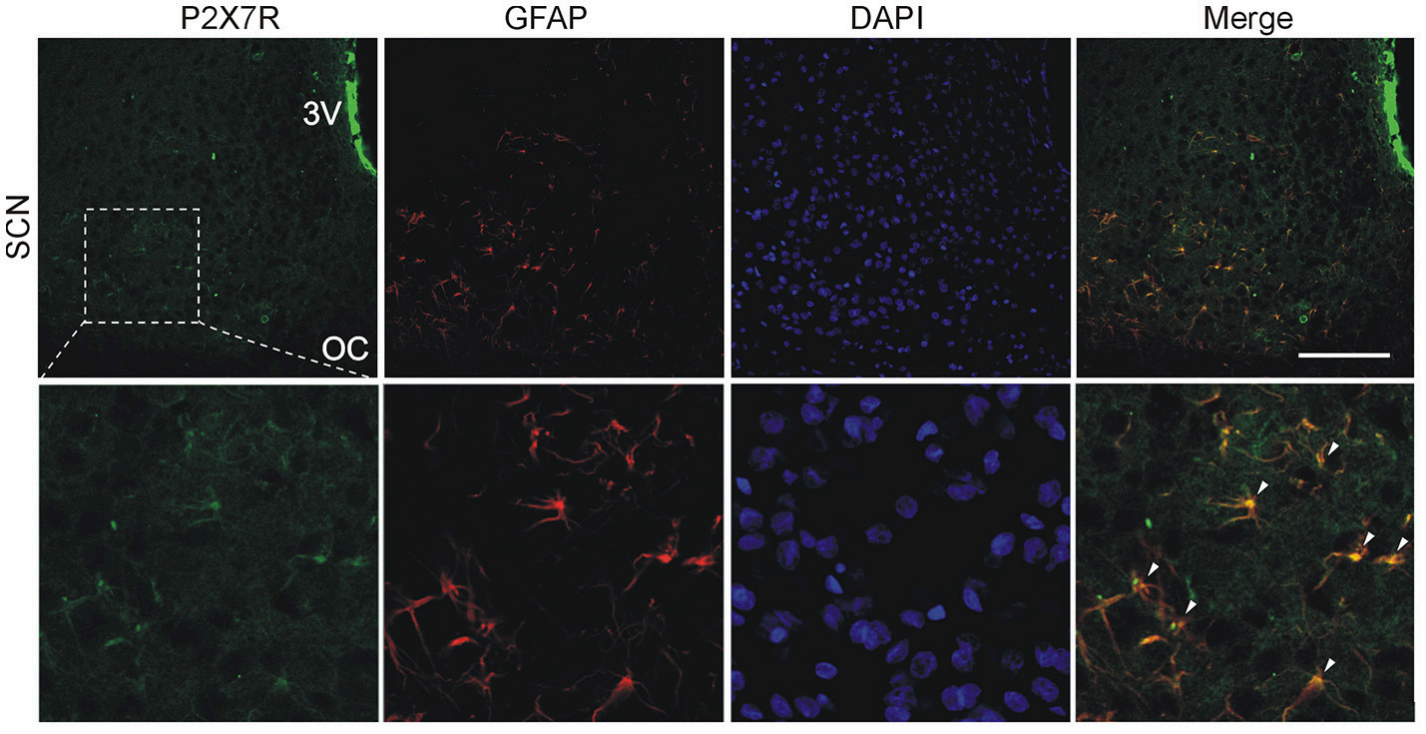
Cellular localization of P2X7Rs in the SCN. Immunohistochemical staining of rat hypothalamic slices containing the SCN, optic chiasm (OC) and the third ventricle (3V). P2X7R immunoreactivity (green) is present on SCN cell somata. DAPI (blue) stains cell nuclei and astrocytes were identified using anti-GFAP antibodies (red). Scale bar: 100 μm **(upper row)**. Colocalization of P2X7R with the astrocyte marker. Arrowheads show representative structures that are double-labeled with anti-P2X7R and anti-GFAP antibodies **(lower row)**. Figure represents example of experiment that was performed on three animals.

We also used double-label immunohistochemistry to examine the expression of P2X7R in combination with the astrocytic marker GFAP. As shown in Figure 4, a relatively high signal for the P2X7R protein colocalized with GFAP was observed throughout the SCN region. Experiments examining the expression of P2XRs in a mixed population of glia cells in 4 days-old culture also showed that astrocytes express P2X7R (Figure S3A), while microglia express mainly P2X4R (Figure S3B) and exhibit much lower expression of P2X7R as compared to astrocytes (Figure S3A).

Thus, P2X7R plays an important role in the circadian accumulation of extracellular ATP in SCN organotypic cultures, and astrocytes represent the source of P2X7R-dependent circadian ATP release.

### Role of pannexin-1 hemichannel in ATP release

**Figure 5:**
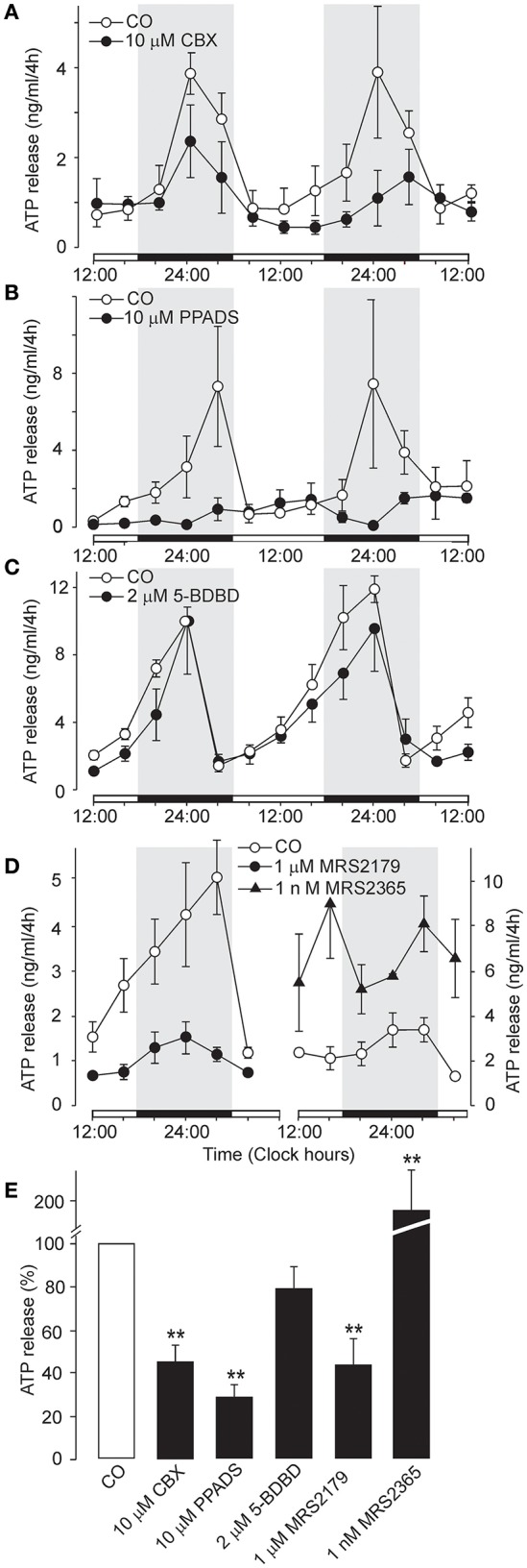
Effects of carbenoxolone and other P2 receptor blockers on the circadian rhythmicity of ATP release. **(A)** Partial inhibitory effect of the pannexin-1 hemichannel blocker carbenoxolone (CBX). **(B)** Inhibition by the non-specific P2 receptor blocker PPADS. **(C)** Lack of an effect of the P2X4R-selective antagonist 5-BDBD. **(D)** Circadian ATP release was significantly decreased by the P2Y1-selective inhibitor MRS2179 (left panel) and potentiated by the P2Y1R-selective agonist MRS2365 (right panel). **(E)** Summary graph showing the effects of the tested drugs on ATP release at the end of the incubation period. Data are presented as the means ± SEM of three to four experiments. ^**^P < 0.01 compared to the control.

We also considered whether the pannexin-1 hemichannel might contribute to extracellular ATP accumulation. Inhibition of astrocytic panneixn-1 hemichannels by carbenoxolone (CBX, 10 μM), which might have also a minor inhibitory effect on connexin hemichannles and gap junction channels (Li M. et al., 2011), reduced the amplitude of the circadian rhythm of ATP release (Figure 5A) and partially inhibited cumulated ATP release (44 ± 9% of the control, *n* = 4, *P* < 0.01; Figure 5E). This data indicate that pannexin-1 hemichannels, alone or in complex with P2X7R, play a role in ATP release.

### Dependence of extracellular ATP accumulation on P2Y1R and P2Y2R activity

In addition to P2X7R, transcripts for P2X1-5 and several P2Y receptors (P2Y1R, P2Y2R and P2Y12R) have also been identified in the SCN (Bhattacharya et al., 2013). The non-selective P2 receptor antagonist PPADS (10 μM) (North, 2002) (Coddou et al., 2011) almost completely inhibited the ATP rhythm (Figure 5B) and extracellular ATP accumulation (28 ± 6% of the control, *n* = 3, *P* < 0.01; Figure 5E), whereas the P2X4R-selective negative allosteric modulator 5-BDBD (2 μM) (Balázs et al., 2013) did not exert a significant effect (79 ± 11%, *P* > 0.05; *n* = 3; Figures 5C,E). Treatment with the P2Y1R-selective antagonist MRS2179 (1 μM) reduced the amplitude of the ATP rhythm (Figure 5D, left panel) and inhibited ATP secretion to 44 ± 12% of control levels (*n* = 5, *P* < 0.01; Figure 5E). In contrast, the P2Y1R-selective agonist MRS2365 (1 nM) potentiated extracellular ATP accumulation (Figure 5D, right panel) to 186 ± 25% of control levels (*n* = 3, *P* < 0.01; Figure 5E). Similar effect was observed with the P2Y2R-selective agonist MRS2768 (0.5 μM; 231 ± 32%, *P* > 0.05; *n* = 2; data not shown). These results showed that, in addition to P2X7R and pannexin-1 hemichannels, metabotropic P2YRs also participate in circadian ATP release.

### Ca^2+^ signals mediated by P2X7 and P2Y receptors in primary cultured SCN astrocytes

**Figure 6:**
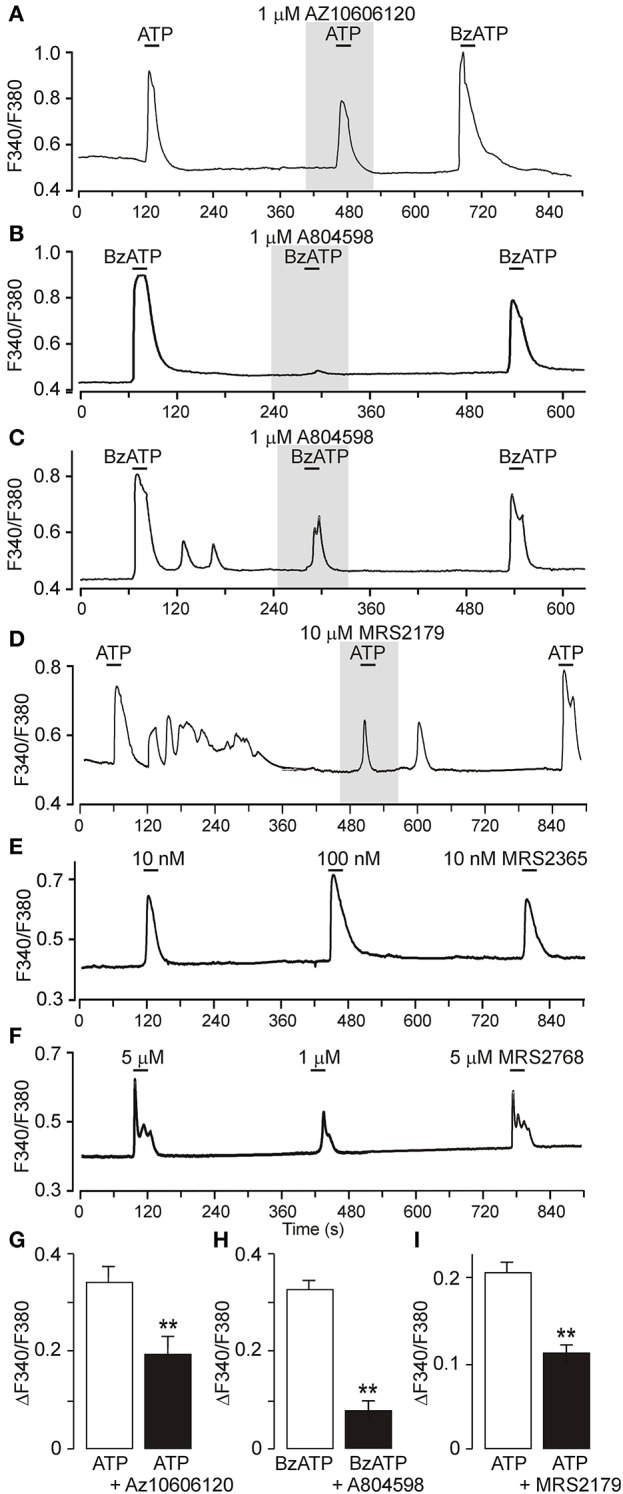
Calcium signaling mediated by P2X7R and P2Y1R in primary cultures of SCN astrocytes. **(A)** Increases in intracellular calcium in cultured SCN astrocytes in response to ATP and BzATP (both 50 μM) and partial inhibition of the ATP-induced [Ca^2+^]_i_ increase by the P2X7R blocker AZ 10606120. The ratio of light intensity (F_340_/F_380_) reflects changes in intracellular calcium concentration ([Ca^2+^]_i_). **(B,C)** BzATP-induced [Ca^2+^]_i_ increases completely **(B)** or partially **(C)** inhibited by the P2X7R blocker A438079. **(D)** Inhibition of ATP-induced responses in the presence of the P2Y1R-selective blocker MRS2179. **(E)** Concentration-dependent elevation of astrocytic calcium levels by the P2Y1R agonist MRS2365. **(F)** Concentration-dependent effect of the P2Y2R agonist MRS2768. **(G–I)** Summary graphs showing the effects of P2 receptor agonists and antagonists on astrocytic [Ca^2+^]_i_. Data are presented as the means ± SEM of three to four experiments. ^**^P < 0.01 compared with the control.

P2Y1R and P2Y2R are coupled to Gq/11 and may thereby contribute to extracellular ATP accumulation by stimulating phospholipase C (PLC) and mobilizing intracellular Ca^2+^ (Abbracchio and Burnstock, 1994). We examined the effects of the P2Y1R agonist MRS2365, the P2Y2R agonist MRS2768, ATP, and the prototypic P2X7R agonist 2′-3′-O-(4-benzoylbenzoyl)-ATP (BzATP) on the intracellular Ca^2+^ concentration ([Ca^2+^]_i_) in primary cultures of SCN astrocytes preincubated with Fura-2AM (*n* = 16 cultures, ~20 cells per culture; Figure 6 and Figure S4). Single cell calcium measurements showed that both ATP and BzATP induced increases in [Ca^2+^]_i_, and the amplitude of the BzATP-induced response was the same as or greater than the ATP-induced responses (Figure 6A), indicating the presence of functional P2X7R. Consistent with this finding, both ATP- and BzATP-induced increases in [Ca^2+^]_i_ were depressed by treatment with the P2X7R-selective blockers AZ10606120 (1 μM, 60 ± 9% inhibition, *n* = 3; Figures 6A,G) and A804598 (1 μM, 25 ± 6% inhibition, *n* = 4; Figures 6B,C,H). ATP-induced responses were also partially reduced by MRS2179 (10 μM, 57 ± 9% inhibition, *n* = 3; Figures 6D,I), indicating involvement of P2Y1R. Application of the P2Y1R-selective agonist MRS2365 elevated [Ca^2+^]_i_ in a concentration-dependent manner (Figure 6E), and a similar effect was observed with the P2Y2R-selective agonist MRS2768 (Figure 6F). Based on these results, both P2X7Rs and P2YRs may stimulate intracellular calcium signals in SCN astrocytes and thereby participate in the Ca^2+^ dependent ATP release.

### Electrophysiological evidence for the lack of an effect of P2X7R and mitochondrial blockers on SCN neuronal activity

**Figure 7:**
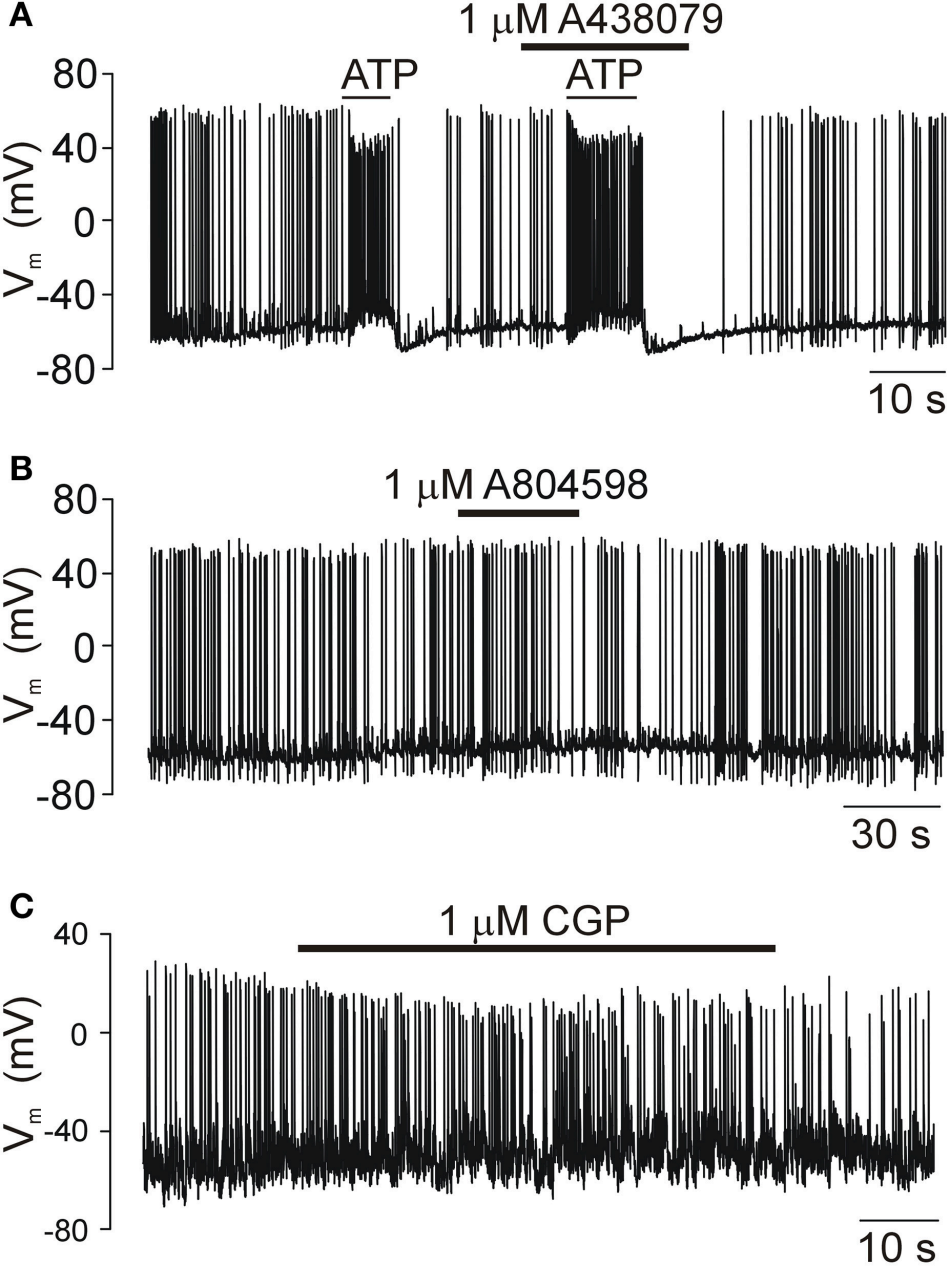
The electrical activity of SCN neurons in acutely isolated slices is not modulated by P2X7R and mitochondria blockers. **(A)** The exogenous ATP application induced an increase in the frequency of action potentials as a result of the increased frequency of depolarizing GABAergic currents caused by the high concentration of chlorides in our intracellular solution (see the section Materials and Methods). The P2X7R-selective inhibitor A438079 had no effect on ATP-induced firing. **(B)** Prolonged application of A804598, another P2X7R blocker, also failed to change the electrical activity of SCN neurons. **(C)** A selective inhibitor of Na^+^-Ca^2+^ exchanger in the mitochondria CGP37157 (CGP), gradually reduced the amplitude of action potentials with no effect on frequency. Traces shown in this figure are representative of 5–6 similar recordings.

We examined the effect of A438079, A 804598, AZ10606120, and CGP 37157 on firing of action potentials by SCN neurons in acutely isolated hypothalamic slices to exclude the possibility that the P2X7R antagonists and blocker of the mitochondrial Na^+^/H^+^ exchanger used in this study might have non-specific effects on ATP release due to their influence on the electrical activity of SCN neurons (Figure 7). Although 100 μM ATP induced an increase in the frequency of action potentials, the preapplication of P2X7R antagonist A438079 had no effect on ATP-induced response (Figure 7A). Application of P2X7R antagonists A438079 (Figure 7B) or AZ10606120 (not shown) was also without any effect. Application of CGP37157 slightly reduced the amplitude of action potentials without any effect on the firing frequency (Figure 7C). These results provide evidence that the inhibitory effects of P2X7R and mitochondrial blockers on ATP release are not associated with the inhibition of neuronal activity.

## Discussion

**Figure 8:**
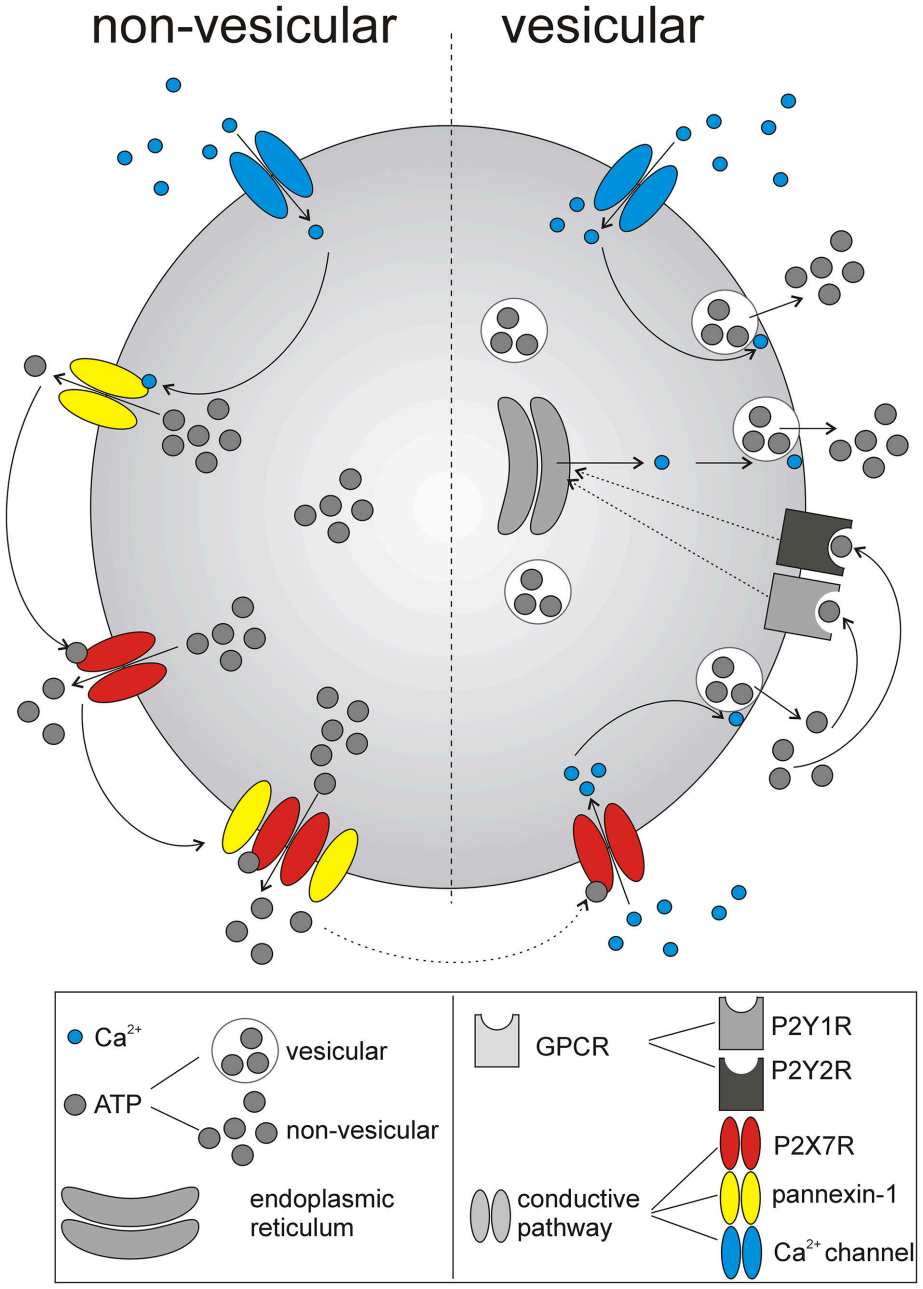
Schematic summary of identified mechanism underlying ATP release from SCN astrocytes. The initial non-vesicular release (predominantly Ca^2+^-independent) of ATP from astrocytes in the SCN is potentially associated with P2X7R and pannexin-1 hemichannel activation. Released ATP further stimulates P2X7R, and G-protein coupled P2YRs (GPCRs), P2Y1Rs, and P2Y2Rs, that evoke Ca^2+^-dependent, vesicular. release of ATP. Thus, multiple purinergic P2 receptor systems, with the contribution of gap junction channels, control release of ATP from SCN astrocytes.

The main finding of our study is that specific P2X7R and P2Y1R antagonists inhibited circadian extracellular ATP accumulation in organotypic SCN cultures, and specific P2X7R, P2Y1R, and P2Y2R agonists elevated intracellular calcium levels in primary cultures of SCN astrocytes, suggesting that both receptors contribute to the circadian rhythmicity of ATP release that might occur via a vesicular or a non-vesicular pathway (Figure 8).

Non-vesicular pathways for ATP release from intact cells may include gap junction proteins like connexins (Stout et al., 2002) or pannexin-1 hemichannels (Schenk et al., 2008; Suadicani et al., 2012) and ATP-gated P2X7R channels (Pellegatti et al., 2005; Hamilton et al., 2008; Nörenberg et al., 2011a). In the brain, both neurons and astrocytes express connexins and form gap junctions, primarily with their own cell type (Contreras et al., 2004). Gap junctional networks of astrocytes are more common and generally more extensive, whereas little evidence is available for gap junctions among SCN neurons (Welsh and Reppert, 1996; Colwell, 2000). The P2X7R protein is also well-documented to be expressed on astrocytes (Narcisse et al., 2005; Sperlágh et al., 2006; Hamilton et al., 2008; Kamatsuka et al., 2014; Zhao et al., 2016), whereas its expression in neurons is still questionable (Illes et al., 2017). Our present study also shows the expression of the P2X7R protein in SCN astrocytes. Together with the ability of the P2X7R and pannexin-1 hemichannel blockers to reduce extracellular ATP accumulation, these data suggest that P2X7R alone or in complex with pannexin may function as a permeation channel for Ca^2+^-independent astrocytic ATP release. Similar functions of P2X7Rs have been proposed for the Ca^2+^-independent release of gliotransmitters, such as purines (Ballerini et al., 1996), GABA (Wang et al., 2002), L-glutamate and D-aspartate (Duan et al., 2003; Cervetto et al., 2013; Di Cesare Mannelli et al., 2015), D-serine (Pan et al., 2015) and ATP (Anderson et al., 2004; Suadicani et al., 2012), from cultured astrocytes. These results may also explain why circadian ATP release in SCN2.2 cells is independent of changes in intracellular Ca^2+^ concentrations (Burkeen et al., 2011) and occurs in cultured mouse cortical astrocytes even after disruption of the vesicular release mechanism (Marpegan et al., 2011).

However, astrocytic [Ca^2+^]_i_ is high in SCN slices during the night (Brancaccio et al., 2017), indicating that Ca^2+^-dependent release of vesicular ATP could also contribute to circadian ATP release. P2X7R activation increases [Ca^2+^]_i_ in rat cerebellar astrocytes (Carrasquero et al., 2009; Nörenberg et al., 2011b; Illes et al., 2012), primary human fetal astrocytes in culture (Narcisse et al., 2005)and rat SCN astrocytes (Bhattacharya et al., 2013). This effect has been also demonstrated to be associated with the P2X7R-dependent and Ca^2+^-dependent release of vesicular ATP (Ballerini et al., 1996; Suadicani et al., 2012) and glutamate (Cervetto et al., 2013) in astrocytes. The involvement of P2Y1R in mediating ATP-evoked Ca^2+^ signals and Ca^2+^-dependent release of vesicular gliotransmiters is well-established in astrocytes (Fumagalli et al., 2003; Verkhratsky et al., 2009). Our finding that ATP release rhythm in SCN organotypic cultures is significantly inhibited by the voltage-gated Ca^2+^ channel blockers is consistent with the possibility that activation of P2X7R produces membrane depolarization and subsequent activation of Ca^2^ entry via voltage-gated Ca^2+^ channels. Finally, increases in [Ca^2+^]_i_ might activate pannexin-1 hemichannels (Stout et al., 2002) which is also relevant for the ATP release through conductive mechanisms.

A P2Y1R agonist (MRS2365), and P2Y2R agonist (MRS2768) increased [Ca^2+^]_i_ and potentiated astrocytic ATP release in our study. However, it is noteworthly that P2Y11R is another possible target for ATP (von Kügelgen and Wetter, 2000). This receptor is also coupled to Gq/11 and has been recently identified in cultured fetal human cortical astrocytes (Muller and Taylor, 2017). However, no selective agonists or antagonists are available yet, and thus the role of P2Y11 in the SCN could not be tested. Metabotropic P2Y1Rs and ionotropic P2X7Rs have been demonstrated to be involved in autocrine stimulation of astrocytic Ca^2+^ signals in intact optic nerves (Hamilton et al., 2008) and astrocyte-to-astrocyte Ca^2+^-mediated communication in culture (Fumagalli et al., 2003). Our data suggest that both P2X7 and P2Y receptors are involved in ATP-induced ATP release from SCN astrocytes, and that the P2X7R-stimulated ATP release is mediated by both Ca^2+^-dependent and Ca^2+^-independent pathway (see scheme, Figure 8).

Our observations raised two important questions. First, we do not know whether the rhythmic ATP secretion from astrocytes normally plays a role in controlling circadian rhythms. Genetically manipulated cortical astrocytes in culture in which IP(3) signaling is inhibited display normal circadian rhythms in clock gene expression despite the presence of arrhythmic extracellular ATP accumulation (Marpegan et al., 2011). Thus, rhythmicity in ATP release is not required for the molecular clockwork. On the other hand, extracellular ATP and its metabolites may function as paracrine modulators in the SCN. We found previously that ATP application modulates the synaptic activity of SCN neurons and via presynaptic P2X2Rs potentiates GABA release (Bhattacharya et al., 2013). Enhanced ATP release could potentially contribute to the low excitability of SCN neurons during the night. Second, further studies are needed to clarify the molecular mechanism by which P2X7 channels are rhythmically activated. Currently, no evidence has been reported suggesting day-night variability in the expression of the P2X7R mRNA or protein in the SCN. Astrocytes respond to many neurotransmitters, but ATP and glutamate are the most prominent (van den Pol et al., 1992; Bhattacharya et al., 2013) and both evoke [Ca^2+^]_i_ signals that might trigger the further release of ATP or glutamate (Haydon, 2001; Illes et al., 2012). Glutamate is released from astrocytes in SCN slices during the night period (Schousboe et al., 2013; Brancaccio et al., 2017), similar to ATP (Yamazaki et al., 1994; Womac et al., 2009). Provided that the glutamate-evoked Ca^2+^-dependent ATP release stimulates the P2X7Rs and the P2YRs, this mechanism could initiate ATP-induced ATP release. Surprisingly, the glutamate-evoked increase in astrocytic [Ca^2+^]_i_ in the intact optic nerve is significantly reduced in P2X7R knock-out mice (Hamilton et al., 2008). However, the control of P2X7R-dependent ATP release by glutamate in SCN astrocytes needs further investigation.

In conclusion, we provide evidence for the involvement of P2X7Rs, P2Y1Rs, and P2Y2Rs in circadian ATP release from astrocytes in SCN organotypic cultures. Purinergic signaling via P2X7Rs is well-known to play important roles in neurodegeneration, neuroprotection and neuroregeneration, and our results might improve our understanding of the roles of these receptors in the healthy central nervous system.

## Author contributions

IS prepared organotypic and primary cultures, performed ATP luciferin-luciferase assays, and contributed to the conception of the work; AB did calcium imaging; MI carried out electrophysiological experiments; ZB performed immunohistochemistry; HZ designed the experiments and wrote the manuscript. All authors analyzed data, contributed to drafting the work, approved the version to be published and agree to be accountable for all aspects of the work.

### Conflict of interest statement

The authors declare that the research was conducted in the absence of any commercial or financial relationships that could be construed as a potential conflict of interest.
