# Supplementary material for: Circadian ATP Release in Organotypic Cultures of the Rat Suprachiasmatic Nucleus Is Dependent on P2X7 and P2Y Receptors
Source: Front Pharmacol. 2018 Mar 6;9:192. doi: 10.3389/fphar.2018.00192 (PMC5845546; doi:10.3389/fphar.2018.00192)
Supplement: Supplementary file 1 [file Image1.pdf]

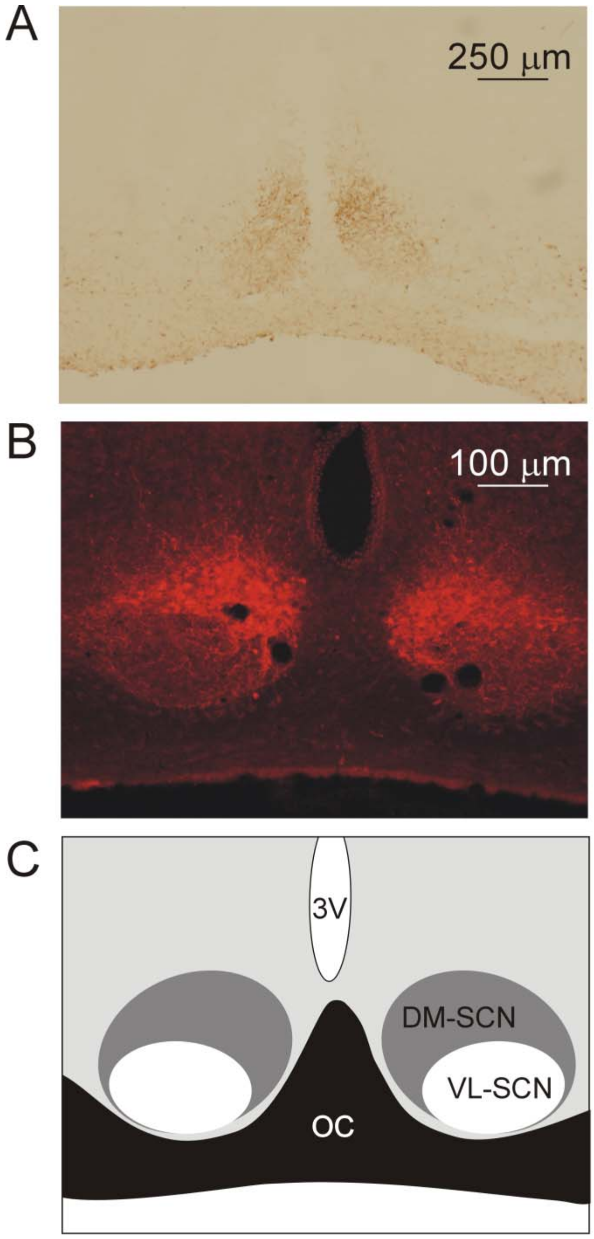

Figure S1

Organotypic cultures and expression of AVP protein in the SCN .

A, An example of immunohistochemistry experiments performed on the rat organotypic cultures containing the SCN. After three days in culture the sections were fixed in 2% paraformaldehyde for 15 min, washed in PBS, and cryoprotected in 20% sucrose in PBS overnight at 4°C. The slices were then frozen on dry ice, sectioned into a 12-lm thick serial sections using a cryostat, and levels of SCN-specific phospho-p44/42 mitogen-activated protein kinase (ERK1/2; antibody purchased from Cell Signaling Technology, Inc., Danvers, MA), were assessed by immunohistochemistry. The immunopositive signal predominates in the dorsomedial subdivision of the SCN. B, Acutely isolated hypothalamic slice containing SCN stained with anti-vasopressin antibody (Abcam - ab39363). The AVP immunoreactivity (red) is present on dorsomedial subdivision of the SON. C, Illusory boundaries showing dorsomedial SCN (DM-SCN), ventrolateral SCN (VL-SCN), optic chiasm (OC) and the third ventricle (3V).
