# Supplementary material for: Circadian ATP Release in Organotypic Cultures of the Rat Suprachiasmatic Nucleus Is Dependent on P2X7 and P2Y Receptors
Source: Front Pharmacol. 2018 Mar 6;9:192. doi: 10.3389/fphar.2018.00192 (PMC5845546; doi:10.3389/fphar.2018.00192)
Supplement: Supplementary file 2 [file Image2.pdf]

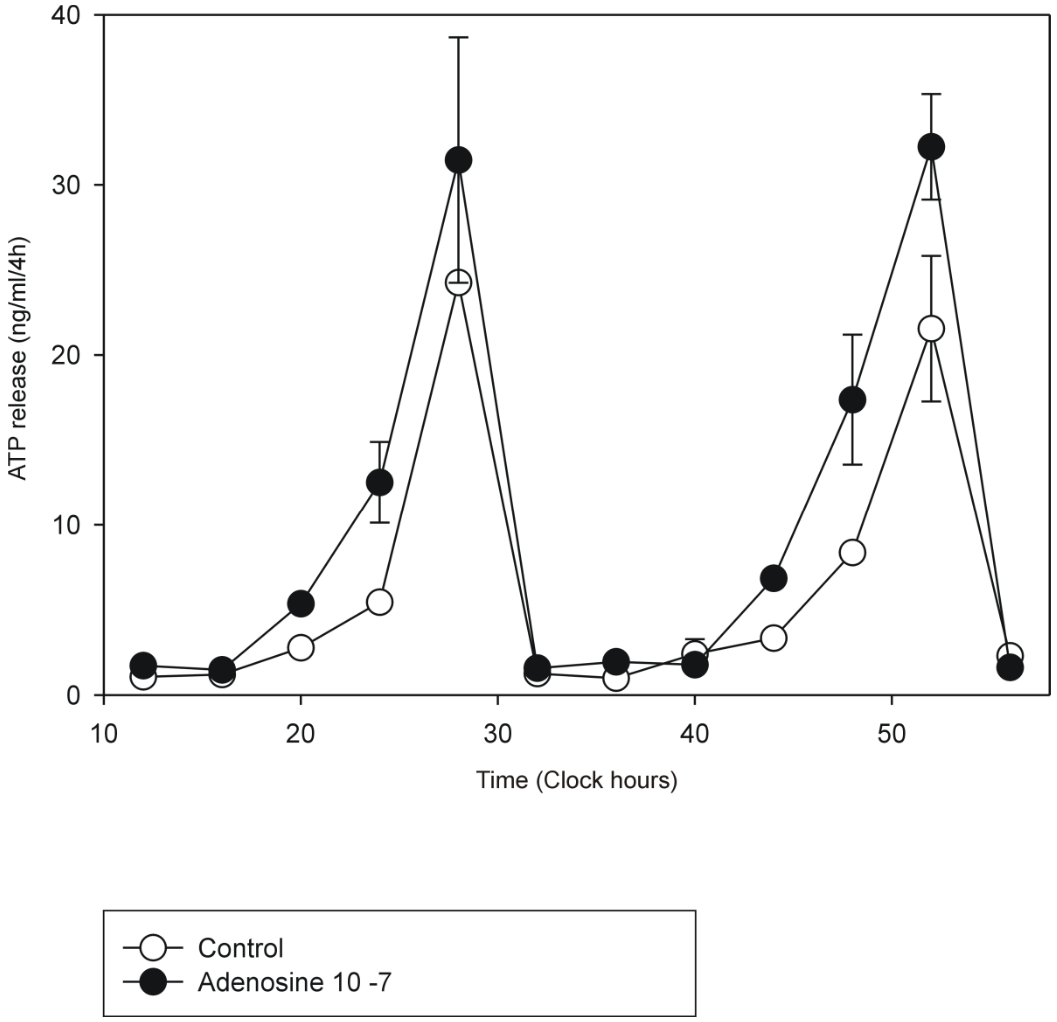

Figute S2

Effect of application of adenosine on ATP release in organotypic slices

Examples of the ATP release rhythm in control cultures (open symbols) and cultures treated with 100 nM adenosine. These results showed that adenosine was not effective inducer of ATP release (124 ± 15% of control, n=3), suggesting that endogenous ATP does not act at adenosine P1 receptors following extracellular metabolism.
