# Supplementary material for: Circadian ATP Release in Organotypic Cultures of the Rat Suprachiasmatic Nucleus Is Dependent on P2X7 and P2Y Receptors
Source: Front Pharmacol. 2018 Mar 6;9:192. doi: 10.3389/fphar.2018.00192 (PMC5845546; doi:10.3389/fphar.2018.00192)
Supplement: Supplementary file 3 [file Image3.pdf]

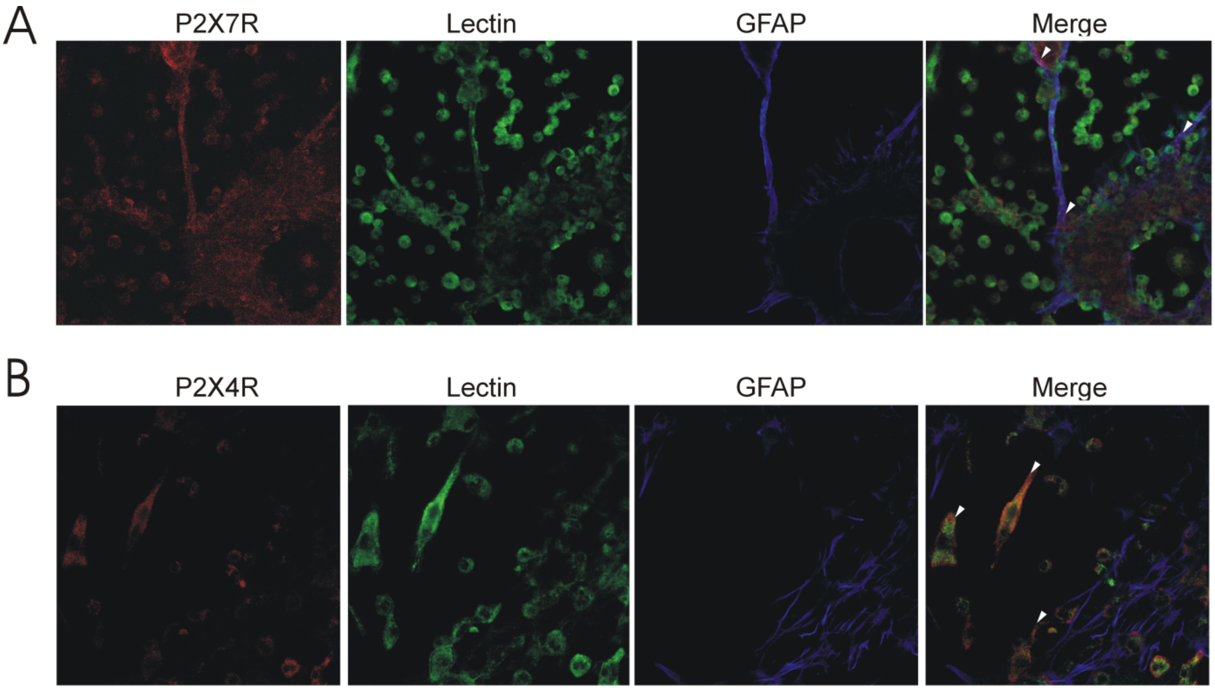

Figure S3

Cellular localization of P2X7R and P2X4R in a mixed population of SCN glia cells in 4-days-old primary culture.

A, Imunohistochemical staining of microglia that were identified using anti-lectin (green) and astrocytes that were identified using anti-GFAP antibodies (blue). Colocalization with P2X7R immunoreactivity (red). B, Colocalization of anti-lectin (green) and anti-GFAP antibodies (blue) with P2X4R immunoreactivity (red). Arrowheads show representative structures that are double-labeled with anti-P2X7R and anti-GFAP antibodies (A, *Merge*) or anti-P2X4R and anti-lectin antibodies (B, *Merge*).
