# Supplementary material for: Circadian ATP Release in Organotypic Cultures of the Rat Suprachiasmatic Nucleus Is Dependent on P2X7 and P2Y Receptors
Source: Front Pharmacol. 2018 Mar 6;9:192. doi: 10.3389/fphar.2018.00192 (PMC5845546; doi:10.3389/fphar.2018.00192)
Supplement: Supplementary file 4 [file Image4.pdf]

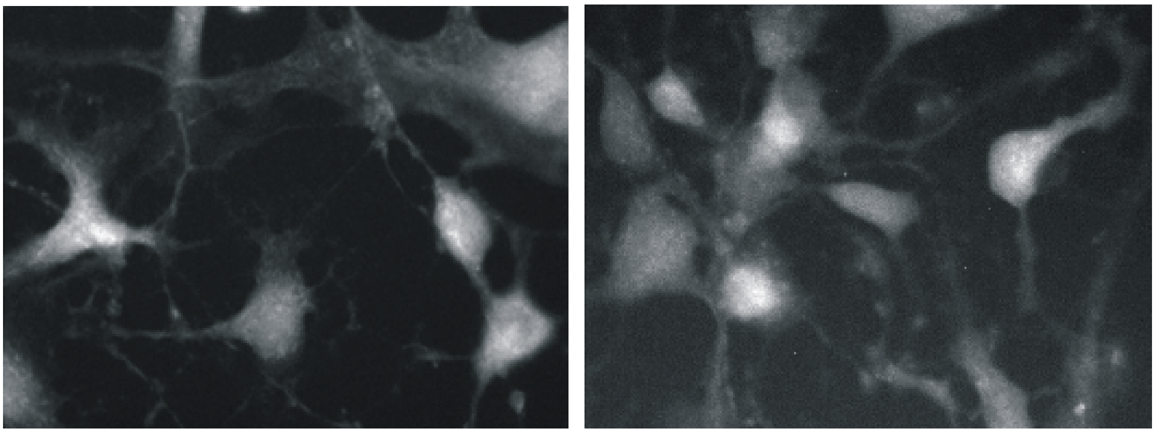

Figure S4

Primary cultures of SCN glia cells used for calcium imaging.

Image of Fura 2AM-stained SCN glia cells in culture (magnification 40x) at 380 nm showing Ca^{2+}-dependent fluorescence in astrocytic structures.
